# Phase-separated stretchable conductive nanocomposite to reduce contact resistance of skin electronics

**DOI:** 10.1038/s41598-024-51980-1

**Published:** 2024-01-16

**Authors:** Hyunjin Lee, Hye Jin Kim, Yoonsoo Shin, Dae-Hyeong Kim

**Affiliations:** 1 Center for Nanoparticle Research, Institute for Basic Science (IBS), Seoul, 08826 Republic of Korea; 2 School of Chemical and Biological Engineering and Institute of Chemical Processes, Seoul National University, Seoul, 08826 Republic of Korea; 3 Department of Materials Science and Engineering, Seoul National University, Seoul, 08826 Republic of Korea

**Keywords:** Polymers, Electrical and electronic engineering

## Abstract

Skin electronics, facilitating a high-quality interface between external devices and human skin for recording physiological and/or electrophysiological signals as well as delivering external electrical and/or mechanical energy into the human body, has shown significant progress. However, achieving mechanically conformal contact and electrically low contact resistance at the device-skin interface remains challenging. Here, we propose a material strategy to potentially address such an issue by using phase separation of silver nanowires and silver nanoparticles (Ag NWs and Ag NPs) within a stretchable conductive nanocomposite (NC). This phase-separated NC ensures low contact resistance and high conductivity, which are key requirements in skin electronics, while maintaining excellent mechanical contact with the skin. To achieve phase separation, we hydrophobically treated the surfaces of Ag NWs and Ag NPs. Then, as the NC solidified, the solvent contained in the NC was slowly evaporated to sufficiently precipitate Ag NPs within the NC. As a result, the phase-separated NC exhibited high conductivity (~ 18,535 S cm^−1^), excellent stretchability (~ 80%), and low contact resistance on both the top and bottom NC surfaces (average ~ 0.132 Ω). The phase-separated NC has enabled implementation of high performance skin-mounted devices, including strain sensors, electrophysiological sensors, and a wearable heater.

## Introduction

Skin electronics, designed to be seamlessly integrated with the soft and curvaceous human skin^1–4^, not only enables the precise recording of physiological and/or electrophysiological signals but also facilitates efficient delivery of external electrical and mechanical energy to the body. This opens up new opportunities in various applications, such as continuous health monitoring, wearable electronics, and soft robotics^5–10^. One of the vital components of these skin electronics is intrinsically stretchable conductors, that should exhibit outstanding electrical as well as mechanical properties. To meet the superior electrical and mechanical performances, such intrinsically stretchable conductors must possess high conductivity, stretchability, skin-like softness, low contact resistance and impedance.

Several types of intrinsically stretchable conductors are available, including gold-deposited elastomers^11–14^, conducting polymers with additives^15–17^, and liquid metal-based elastomers^18–20^. Despite recent advances, these materials still face remaining challenges. Gold-deposited elastomers exhibit high conductivity and excellent biocompatibility. However, under deformation, their conductivity significantly decreases due to extensive crack propagation^21^. Conducting polymers exhibit excellent electrochemical properties with low impedance^22^. Nonetheless, they possess relatively low conductivity and stretchability, and they are susceptible to dehydration under atmospheric conditions. Liquid metal-based elastomers show remarkable conductivity, strain-insensitive resistance, and high stretchability^23^. Still, the potential for leakage and oxidation of liquid metal restricts their use in skin electronics.

One of promising materials for intrinsically stretchable conductors is stretchable conductive nanocomposites that incorporate metal fillers^24–28^. Various metal fillers can be considered, among which silver nanowires (Ag NWs) have been widely used, since they possess high intrinsic conductivity (~ 630,000 S cm^−1^)^29^ and feature high aspect ratios that enable the formation of a dense electrical percolation network. These characteristics allows for the realization of stretchable nanocomposites with high conductivity and stretchability^30–37^. However, the contact resistance of the nanocomposite to the skin still exhibits unsatisfactorily high values, since the majority of Ag NWs are embedded within the insulative elastomeric matrix, while only a few Ag NWs that make electrical connection to the skin are exposed on the surface of the matrix^38^. Along with the high contact resistance, the charge transfer at the interface between the nanocomposite and the skin or other electrodes is restricted, ultimately leading to inaccurate signal recording and low signal transmission efficiency^39–41^. Therefore, new material strategies are needed to reduce the contact resistance of nanocomposites while maintaining high conductivity and stretchability.

Herein, we present a material strategy to fabricate a high-performance nanocomposite (NC) which satisfies the ideal material properties such as high conductivity, stretchability, and low contact resistance, simultaneously. Such high-performance NC is achieved through phase separation of Ag NWs and silver nanoparticles (Ag NPs) within the NC. To achieve the phase separation, both surfaces of Ag NWs and Ag NPs were treated to become hydrophobic, and the evaporation speed of solvent within the NC was controlled to be slow enough for sufficient phase separation. Consequently, the phase-separated NC exhibits remarkable properties, including a high conductivity of ~ 18,535 S cm^−1^, a reasonable stretchability of ~ 80%, and a low contact resistance of ~ 0.132 Ω. The performance of the phase-separated NC was effectively demonstrated through various skin-mounted devices. These include strain sensors capable of accurately detecting and quantifying mechanical deformations, as well as electrophysiology sensors for precise measurement of electrical signals in biological systems. Furthermore, the phase-separated NC was employed as a wearable heater, showcasing its ability to efficiently convert electrical energy into heat. These diverse applications highlight the versatility and potential of the phase-separated NC in various fields, ranging from healthcare and biotechnology to robotics and beyond.

## Results

### Phase separation of Ag NWs and Ag NPs within the NC

**Figure 1:**
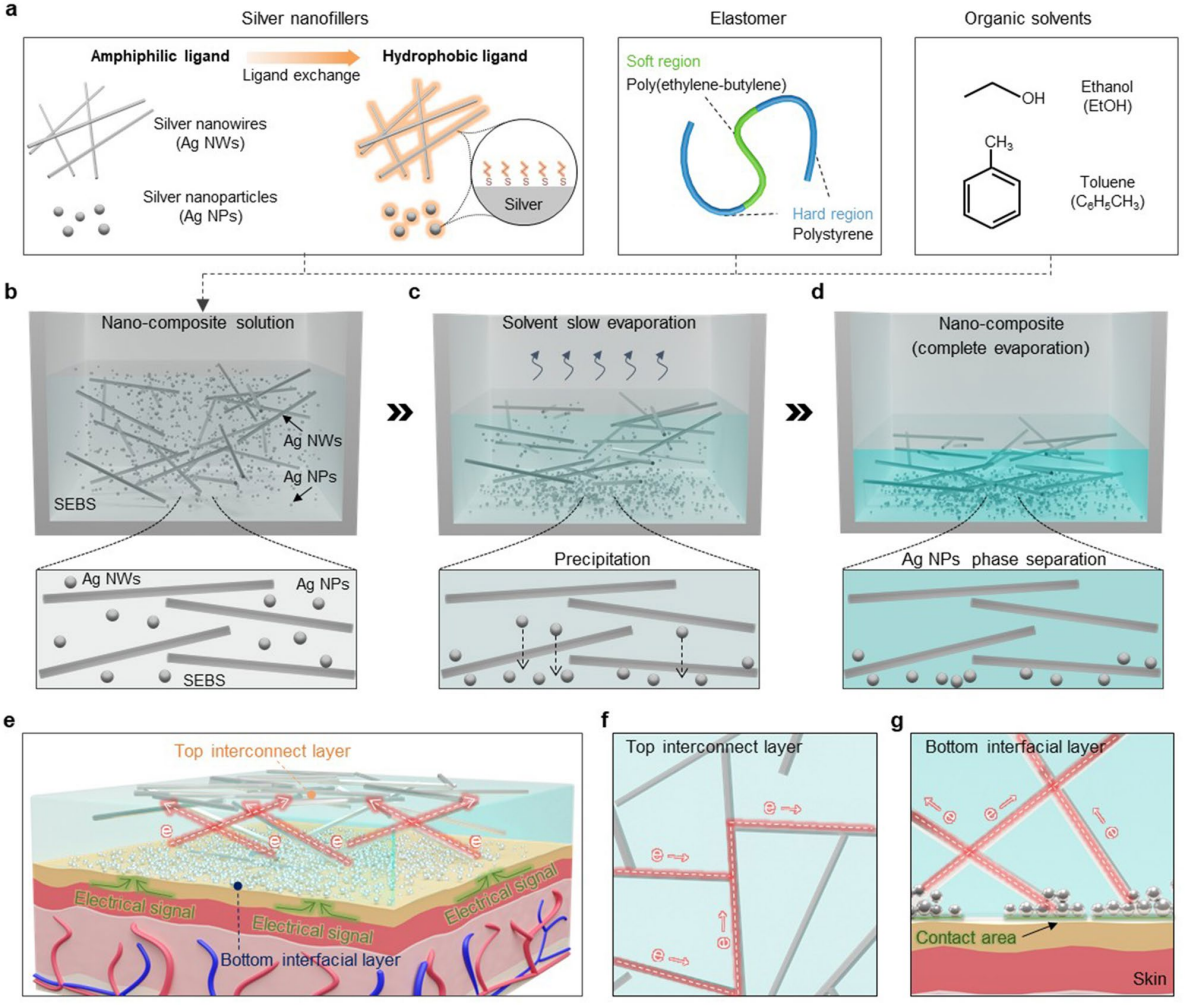
Fabrication process of the phase-separated NC. (**a**) Schematic illustration depicting the constituents of the NC solution. (**b**)**-**(**d**) Sequential schematic illustrations showing the precipitation of Ag NPs during the slow evaporation process. Initial mixed state of Ag NWs and Ag NPs (**b**). Precipitation of Ag NPs during solvent evaporation (**c**). Phase separation of Ag NPs within the solidified NC after complete solvent evaporation (**d**). (**e**)**–**(**g**) Schematic illustration of the phase-separated NC. Complete section of the NC affixed on the skin (**e**). Top surface of the NC with Ag NWs network (**f**). Bottom surface of the NC, where the precipitated Ag NPs make contact between the Ag NWs and the skin (**g**).

NC is produced via the drop-casting method of a solution composed of Ag nanomaterials, poly(styrene-ethylene-butylene-styrene) (SEBS) elastomer, and organic solvents (ethanol and toluene) (Fig. 1a)^42^. To induce phase separation within the NC, two types of Ag nanomaterials, Ag NWs and Ag NPs, are used and both surfaces are modified to be hydrophobic through ligand exchange. Ag NWs and Ag NPs were synthesized using previously reported methods^33,34^. The synthesized Ag NWs have a diameter and length of ~ 211 nm and ~ 80 µm, respectively, while the synthesized Ag NPs have a diameter of ~ 90 nm (Fig. S1a-c).

When a NC solution that contains only Ag NWs is poured into a Teflon mold, the SEBS matrix gradually solidifies, starting from the bottom of the mold as the organic solvent evaporates (Fig. S2a). During the initial solidification stage, the SEBS solution spreads uniformly on the bottom of the mold, embedding most of the Ag NWs within the SEBS matrix. As the solidification proceeds, Ag NWs form a percolation network within the SEBS matrix, and some Ag NWs protrude from the matrix (Fig. S2b). Finally, when the SEBS is completely solidified, Ag NWs on the lower surface are mostly embedded within the SEBS matrix, while on the upper surface, some Ag NWs protrude externally and are exposed out of the top surface. As a result, on the upper surface of the NC, charge transfer to the external media (e.g., human skin) can occur through the protruding Ag NWs, but on the lower surface, where most Ag NWs are embedded within the SEBS matrix, charge transfer to the external material is relatively restricted (Fig. S2c,d). This leads to a difference of the contact resistance between the top and bottom surfaces of the NC (Fig. S2e,f). Note that the Ag NPs observed on the bottom surface of NC are residues generated during the synthesis of Ag NWs.

On the other hand, when a NC solution containing both Ag NWs and Ag NPs with hydrophobically treated surfaces is poured into the mold, the Ag NWs and Ag NPs exhibit different behaviors. Initially, both Ag NWs and Ag NPs are dispersed homogeneously in the solution without aggregation (Fig. 1b). As the organic solvent gradually evaporates at room temperature and SEBS slowly solidifies, the Ag NWs are distributed within the solidifying SEBS matrix. Meanwhile, interestingly, the Ag NPs gradually sink downward within the solidifying SEBS matrix (Fig. 1c). Ultimately, upon the complete solidification of SEBS, the Ag NPs precipitate on the bottom surface of the NC, resulting in phase separation within the NC (Fig. 1d). Note that the thickness of NC was ~ 23.57 μm (Fig. S1d). The phase separation facilitates effective charge transfer on both the upper and lower surfaces of the NC (Fig. 1e). On the upper surface of the phase-separated NC, edge parts of some Ag NWs are exposed out of the SEBS matrix (Fig. 1f), thus the charge transfer on the upper surface of the NC occurs through the exposed ends of the Ag NWs. On the lower surface of the NC, the precipitated Ag NPs contacted to the Ag NWs lead to effective charge transfer (Fig. 1g). As a result, the phase-separated NC exhibits low contact resistance on both the top and bottom surfaces, enabling efficient transmission of electrical signals. Additionally, the phase-separated NC patterned with a laser cutter maintains its stretching performance without experiencing mechanical fractures even at an applied strain of 50% (Fig. S3).

### Surface modification of Ag NWs and Ag NPs for the phase separation

The surface properties of Ag NWs and Ag NPs determine the aggregation between them, enabling the control of their phase separation within the NC. As-synthesized Ag NWs and Ag NPs typically possess poly(vinylpyrrolidone) (PVP) ligands on their surfaces. To investigate the effect of surface properties, synthesized Ag NWs and Ag NPs are treated with 400 μL of 3-Mercapto-1-propanol (C_3_OH) and 1-Propanethiol ligands (C_3_SH) solutions for 2 h to make their surfaces more hydrophilic and hydrophobic, respectively. After surface modification, the surface properties of Ag NWs and Ag NPs were confirmed through thermogravimetry analysis (TGA) (Fig. S4a). Detailed information regarding the surface modification and analysis of Ag NWs and Ag NPs is described in the Methods section.

**Figure 2:**
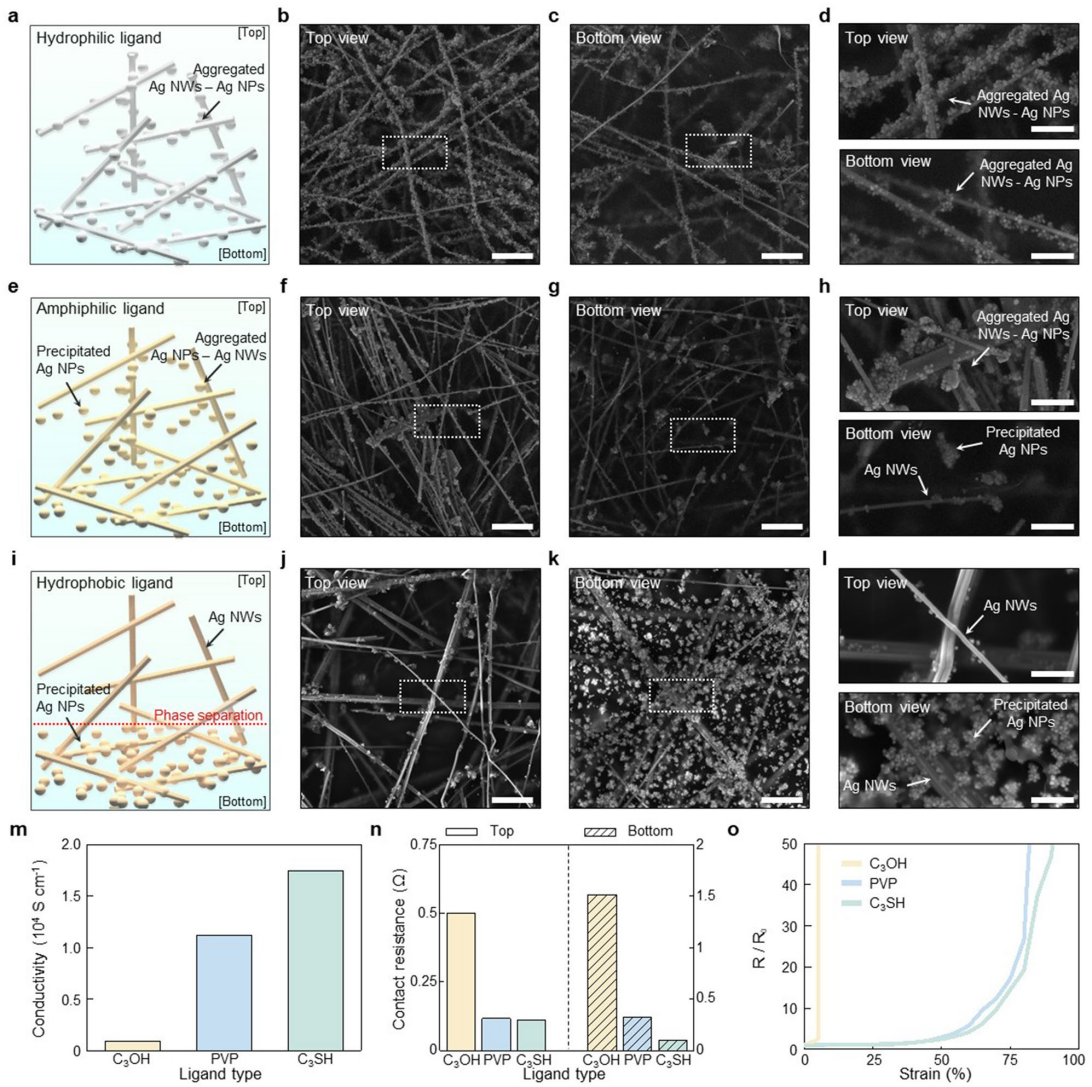
Materials performances of phase-separated NC depending on the ligand types. (**a**)-(**d**) NC composed of hydrophilic Ag NWs and Ag NPs. Schematic illustration inside the NC (**a**). SEM images showing both the top and bottom surfaces of the NC (**b**-**d**). (**e**)–(**h**) NC composed of amphiphilic Ag NWs and Ag NPs. Schematic illustration inside the NC (**e**). SEM images showing both the top and bottom surfaces of the NC (**f**–**h**). (**i**)-(**l**) NC composed of hydrophobic Ag NWs and Ag NPs. Schematic illustration inside the NC (**i**). SEM images showing both the top and bottom surfaces of NC (**j**-**l**). (**m**) Conductivities of NCs according to ligand types. (**n**) Contact resistances of the top and bottom surfaces of NCs by ligand types. (**o**) Stretchabilities of NCs according to ligand types. Scale bars, 3 μm (**b**, **c**, **f**, **g**, **j**, **k**); 1 μm (**d**, **h**, **l**).

When the surfaces of Ag NWs and Ag NPs were modified with C_3_OH, they become hydrophilic (Fig. S4b,c; Table S1). The hydrophilicity causes strong aggregation with each other, resulting in the majority of the Ag NPs to adhere to the surface of the Ag NWs (Fig. 2a). As a result, the Ag NPs do not precipitate into the lower region of the SEBS matrix, prohibiting the phase separation between the Ag NWs and Ag NPs. It has been confirmed by observing the top and bottom surfaces of the NC using scanning electron microscopy (SEM). In both the top and bottom surfaces, most Ag NPs are attached to the surface of Ag NWs (Fig. 2b–d). In contrast, Ag NWs and Ag NPs with PVP ligands manifest amphiphilic property (Fig. S4d, e; Table S1), resulting in fewer Ag NPs adhering to the surface of the Ag NWs. Consequently, unattached Ag NPs precipitate to the lower region of the SEBS matrix (Fig. 2e). Nonetheless, due to the majority of Ag NPs remain adhered to the surface of the Ag NWs, substantial precipitation of the Ag NPs barely occurs. SEM images on the top surface of the NC show the Ag NWs on which some Ag NPs are attached (Fig. 2f,h), and such Ag NWs and precipitated Ag NPs can be observed at the bottom surface of the NC (Fig. 2g,h).

Meanwhile, the surface modification of Ag NWs and Ag NPs with C_3_SH ligands imparts hydrophobicity to their surfaces (Fig. S4f., g; Table S1). This hydrophobicity prevents the Ag NPs from adhering to the surface of Ag NWs, thus causing the majority of the Ag NPs to move downward and precipitate within the SEBS matrix (Fig. 2i). As a result, the Ag NWs are uniformly distributed within the NC, while the majority of the Ag NPs are located at the bottom of the NC—that is, phase separation occurs within the NC. Indeed, SEM images on the top (Fig. 2j,l) and bottom (Fig. 2k,l) surfaces of the NC reveal that the Ag NPs barely adhered to the Ag NWs surface but they mostly precipitated at the bottom. These results demonstrate that as the surfaces of Ag NWs and Ag NPs become more hydrophobic, the phase separation of Ag NPs within the NC becomes more promoted.

Phase separation within the NC enhances conductivity of the NC and improves charge transfer efficiency at its bottom surface. The conductivities of different types of NCs are ~ 914 S cm^−1^ (NC with C_3_OH ligand), ~ 11,220 S cm^−1^ (NC with PVP ligand) and ~ 17,478 S cm^−1^ (NC with C_3_SH ligand), respectively (Fig. 2m). The charge transfer efficiency is estimated by measuring contact resistance of the top and bottom surfaces of the NC. For the NC with C_3_OH ligand, both the top and bottom contact resistances are relatively high, ~ 0.501 Ω and ~ 1.512 Ω, respectively. However, as phase separation occurs, contact resistances decrease significantly to ~ 0.116 Ω and ~ 0.324 Ω in the NC for PVP ligand, and ~ 0.111 Ω and ~ 0.101 Ω for the NC with C_3_SH ligand, respectively (Fig. 2n). Furthermore, the NC with C_3_OH ligand displays limited stretchability of less than 10%, whereas both NCs with PVP ligand and C_3_SH ligand exhibit reasonable stretchability (up to ~ 80%) (Fig. 2o). The improved stretchability observed in the phase-separated NC is caused by the complementary role of the electrical percolation networks formed by the phase-separated Ag NWs and Ag NPs. In the case of effective phase separation, the electrical percolation network formed by Ag NWs is evenly distributed throughout the NC, and the presence of Ag NPs on the bottom surface reinforces this network (Fig. S5a). Consequently, this enhanced percolation network plays a crucial role in minimizing disruptions under strain, thereby contributing to the exceptional stretchability of the NC. Conversely, when effective phase separation does not occur, the electrical percolation network within the NC is solely reliant on Ag NWs, resulting in partial breaks under external strains and an increase in resistance (Fig. S5b). At a 50% applied strain, resistance variation (i.e., R/R_0_) for the NC with PVP ligand is approximately 2.92, while it is reduced to 2.45 for the NC with C_3_SH ligand. Also, we assessed the durability and long-term performance by measuring the resistance change of the NC under repetitive 30% strain. As a result, the phase-separated NC exhibited stable resistance change throughout repeated stretching, contrasting with the unstable resistance change observed in the original NC from the initial stages of stretching (Fig. S5c,d). These results demonstrate that the phase separation between Ag NWs and Ag NPs within the NC not only enhances conductivity and reduces contact resistance but also maintains electrical stability under repetitive stretching conditions.

### Maximizing precipitation of Ag NPs via solvent evaporation control

**Figure 3:**
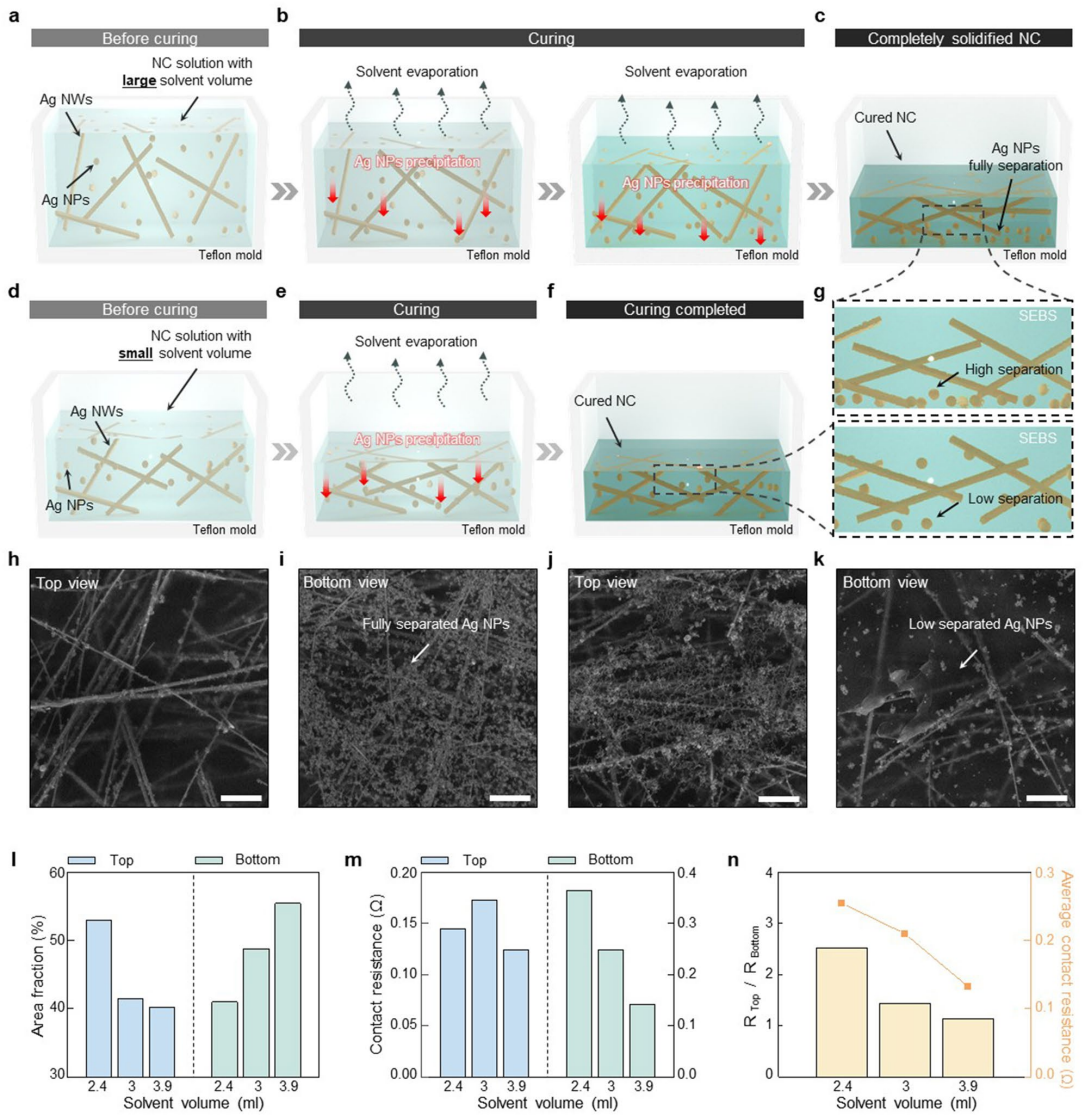
Material performance of the phase-separated NC depending on the solvent evaporation speed. (**a**)–(**c**) Schematic illustrations showing the slow solvent evaporation in the NC solution containing a large amount of solvent. (**d**)**–**(**f**) Schematic illustrations showing rapid solvent evaporation in the NC solution containing a small amount of solvent. **g**, Schematic illustrations for magnified views of **c** and **f**. **h–k** SEM images of both the top and bottom surfaces of the NCs. NC produced with 3.9 mL of solvent (**h**, **i**). NC produced with 2.4 mL of solvent (**j**,**k**). **l** Area fractions of Ag NWs and Ag NPs on the top and bottom surfaces of various NCs, respectively. (**m**) Contact resistances of the top and bottom surfaces of NCs according to the solvent volume (Contact with copper (Cu) foil, contact area = 14 mm^2^). (**n**) Relative contact resistances (R_Top_/R_Bottom_, left) and average contact resistances (right) depending on the solvent volume. Scale bars, 3 μm (**h–k**).

Phase separation within NC is affected by the time required for complete solvent evaporation from a NC solution. To demonstrate the relation between phase separation and solvent evaporation speed, we manipulated the quantity of solvent in the NC solution. When the amount of solvent present in the solution is large, it takes a significant amount of time for the solvent to evaporate completely (Fig. 3a). This long evaporation time ensures Ag NPs to precipitate sufficiently within the SEBS matrix (Fig. 3b). Consequently, upon complete solvent evaporation, distinct phase separation occurs, with Ag NPs only occupying the bottom surface of the NC (Fig. 3c,g). Conversely, when the quantity of solvent in the NC solution is low, the solvent evaporation completes in a relatively short period of time (Fig. 3d). Due to this short evaporation time, Ag NPs are not adequately precipitated within the SEBS matrix (Fig. 3e). Therefore, when solvent evaporation is completed, the phase separation within the NC is less than the case with higher solvent amounts (Fig. 3f,g).

To investigate the effect of the solvent evaporation speed in more detail, we adjusted the quantity of solvent in the NC solution to 2.4 mL, 3.0 mL, and 3.9 mL, and subsequently allowed it to completely evaporate at room temperature. The NC solution is composed of equal amounts of Ag NWs (30 mg), Ag NPs (15 mg), and SEBS (500 μL), with the solvent ratio of ethanol to toluene maintained consistently at 1:5. The NC containing 3.9 mL of solvent clearly demonstrates distinct phase separation. SEM images of the top and bottom surfaces of the NC reveal uniformly dispersed Ag NWs (Fig. 3h) and the precipitated Ag NPs (Fig. 3i), respectively. In contrast, in the case of the NC containing 2.4 mL of solvent, less phase separation is observed. More aggregation between the Ag NWs and Ag NPs on the top surface is noticeable (Fig. 3j), accompanied by reduced precipitation of Ag NPs on the bottom surface (Fig. 3k), compared to the NC with a higher amount of the solvent. SEM images of the top and bottom surfaces of the NC with the solution that contains 3.0 mL of the solvent are shown in the supplementary information (Fig. S6a,b).

The distribution of Ag NWs and Ag NPs on the top and bottom surfaces of the NC are quantified using Image J. As the amount of solvent within the NC solution increases from 2.4 mL to 3.0 mL, and to 3.9 mL, the area fraction of Ag NWs on the top surfaces of the NCs decreases from ~ 53% to ~ 41%, and to ~ 40%, respectively. Conversely, with the increase of the solvent content within the NC solution, the area fraction of Ag NPs on the bottom surfaces of the NCs rises from ~ 40% to ~ 48%, and to ~ 55%, respectively (Fig. 3l). These results imply that slow solvent evaporation effectively facilitates the phase separation within the NC. The phase separation within the NC leads to decrease of contact resistance on both the top and bottom surfaces of the NC. For the solvent quantities of 2.4 mL, 3.0 mL, and 3.9 mL within the NC solution, the corresponding contact resistances on the top and bottom surfaces of the NC are ~ 0.145 Ω, ~ 0.172 Ω, ~ 0.124 Ω, and ~ 0.365 Ω, ~ 0.247 Ω, ~ 0.141 Ω, respectively (Fig. 3m). With the more phase separation, the less difference in contact resistance between the top and bottom surfaces of the NC is observed, which also leads to the lower average resistance (Fig. 3n).

### Performance comparison of the NC with and without phase separation

**Figure 4:**
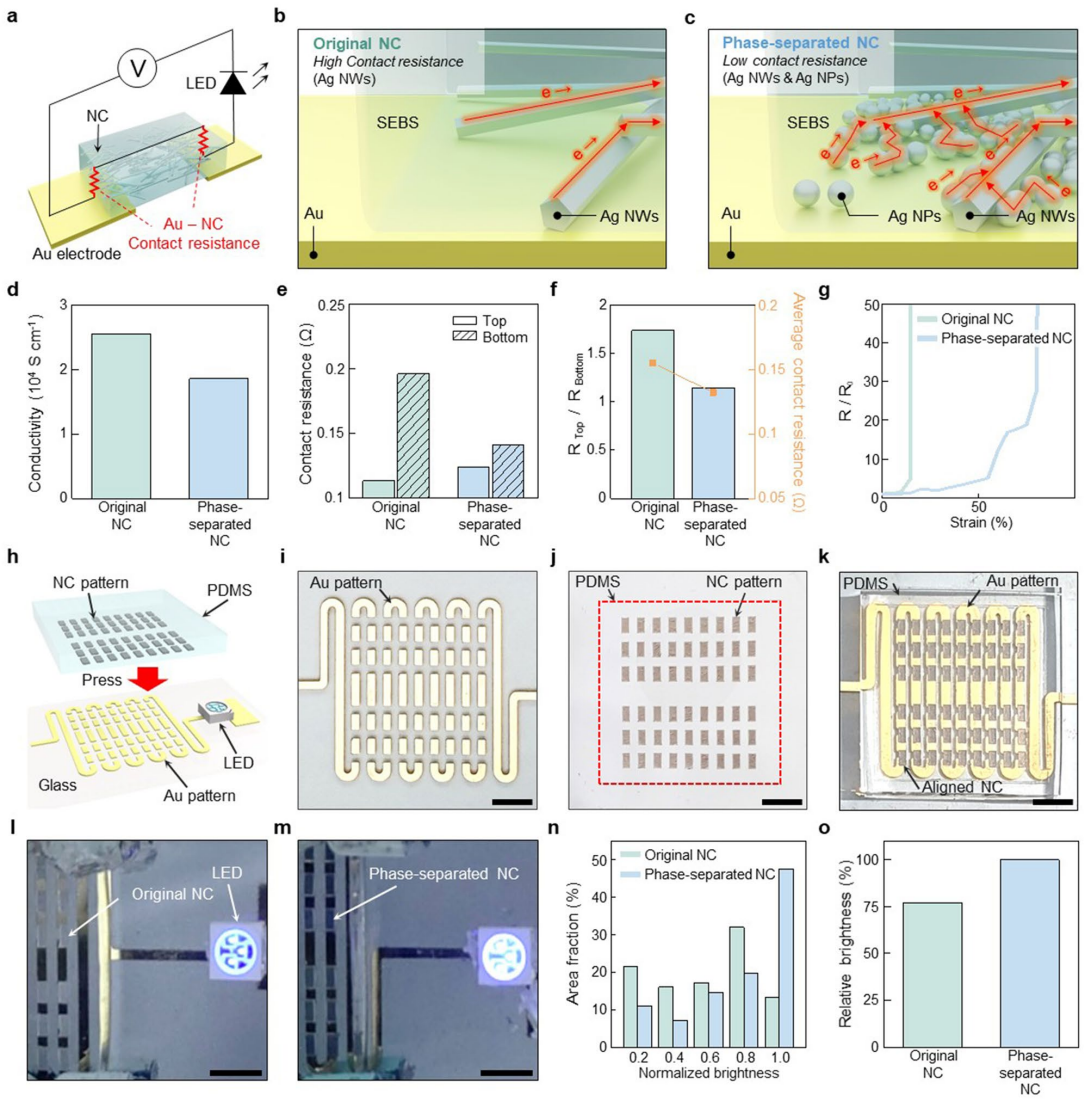
Performance comparison of the NC with and without phase separation. (**a**) Schematic illustration showing the contact resistance between Au electrodes and the NC. (**b**), (**c**) Detailed illustration showing the contact between Au electrodes and NC: the original (**b**) and the phase-separated NCs (**c**). (**d**)**-**(**g**) Electrical performances of the original and phase-separated NCs: Conductivities (**d**), Contact resistances of the top and bottom surfaces (**e**), Relative (left) and average contact resistances (right) of the top and bottom surfaces of NCs (**f**), and Stretchabilities (**g**). (**h**) Schematic illustration showing a LED circuit with NC array patterns attached to the Au patterns. (**i**)**-**(**k**) Optical images of the Au patterns on a glass substrate (**i**), the NC array patterns on a PDMS substrate (**j**) and the aligned NC array patterns on the Au patterns for electrical connection (**k**). (**l**), (**m**) Optical images of the LED circuit with the original and phase-separated NCs. (**n**) Normalized brightness of the LED with corresponding area fraction. (**o**) Relative brightness of the LED with the original and the phase-separated NCs. Scale bars, 5 mm (**i-m**).

We compared the electrical performance of two types of NC: one is composed solely of Ag NWs without phase separation (referred to as “original NC” hereafter) and the other is a phase-separated NC. The original NC consists of 54 wt% Ag NWs, whereas the phase-separated NC contains 36 wt% Ag NWs and 18 wt% Ag NPs. The amount of SEBS and solvent contained in both types of NCs are same as 40 mg and 3.9 mL respectively. These two types of NC are placed on gold (Au) electrodes of which contact resistance is analyzed (Fig. 4a).

In the original NC, the majority of Ag NWs are uniformly distributed within the SEBS matrix, with only a few Ag NWs protruding from the top surface of the SEBS matrix. On the lower surface, most of the Ag NWs are embedded inside the SEBS matrix, resulting in poor charge transfer efficiency through the bottom surface (Fig. 4b). Therefore, there is a substantial difference of contact resistance between the top and bottom surfaces of the original NC, among which the bottom contact resistance is higher. Conversely, in the case of the phase-separated NC, the precipitated Ag NPs promote charge transfer efficiency on the bottom surface of the NC. Consequently, the efficient charge transfer is achieved on both the top and bottom surfaces of the phase-separated NC, leading to reduced contact resistance on the bottom side (Fig. 4c).

The performance of these two types of NCs is validated by comparing their conductivity, contact resistance, and stretchability. The conductivities of the original and phase-separated NCs are ~ 25,456 S cm^−1^ and ~ 18,535 S cm^−1^, respectively (Fig. 4d). The higher conductivity of the original NC than the phase-separated NC can be attributed to the formation of a denser percolation network by Ag NWs than Ag NPs with the same mass fraction. The contact resistances of the top and bottom surfaces are ~ 0.113 Ω and ~ 0.196 Ω, and ~ 0.124 Ω and ~ 0.141 Ω, for the original and phase-separated NCs, respectively (Fig. 4e). As previously mentioned, in the original NC, a portion of Ag NWs is exposed on the upper surface, leading to low contact resistance. However, on the lower surface of the original NC, most of the Ag NWs are embedded within the SEBS matrix, resulting in a high contact resistance. Conversely, in the phase-separated NC, both Ag NWs exposed on the top surface and Ag NPs precipitated at the bottom surface contribute to low contact resistance of both surfaces of the NC. Thus, the top and bottom contact resistances of the phase-separated NC are similar as well as low, making it more suitable for interfacing with other materials than the original NC (Fig. 4f). The difference in contact resistances between the top and bottom surfaces of the NC is determined by dividing the measured contact resistance values on both surfaces, which are ~ 1.73 and ~ 1.14 for the original and the phase-separated NCs, respectively. Additionally, the average contact resistances of original and phase-separated NCs are ~ 0.155 Ω and ~ 0.132 Ω, respectively. In terms of stretchability, the phase-separated NC exhibits higher stretchability than the original NC, indicating its suitability for use as soft skin electronics (Fig. 4g).

The difference in the electrical performances of the two types of NCs is validated through their implementation to a simple circuit for a light emitting diode (LED) (Fig. 4h). The brightness of the LED decreases as resistance increases, making it a useful parameter for comparing the performance of the original and phase-separated NCs. To implement the LED circuit, NCs patterned with a laser cutter were situated between Au electrodes patterned on a glass substrate through photo-lithography, and LED was attached to the ends of the connected Au-NC pattern (Fig. 4i-k). Under a constant voltage of 2.3 V, the brightness of the LED using the phase-separated NC interconnection is brighter than that of the original NC (Fig. 4l,m). We quantified the LED brightness on a per-pixel basis, categorized into 10 levels, and then presented the area fraction of the corresponding pixels for each brightness level (Fig. 4n). In the original NC, the broadest area corresponds to the brightness of 0.8, while for the phase-separated NC, the widest area represents the brightness of 1.0. In terms of average brightness, the phase-separated NC outperforms the original NC, with a ratio of ~ 76:100 (Fig. 4o). This indicates that the phase-separated NC with significantly reduced contact resistance contributes to the heightened LED brightness.

### Application of the stretchable conductive nanocomposite to skin electronics

**Figure 5:**
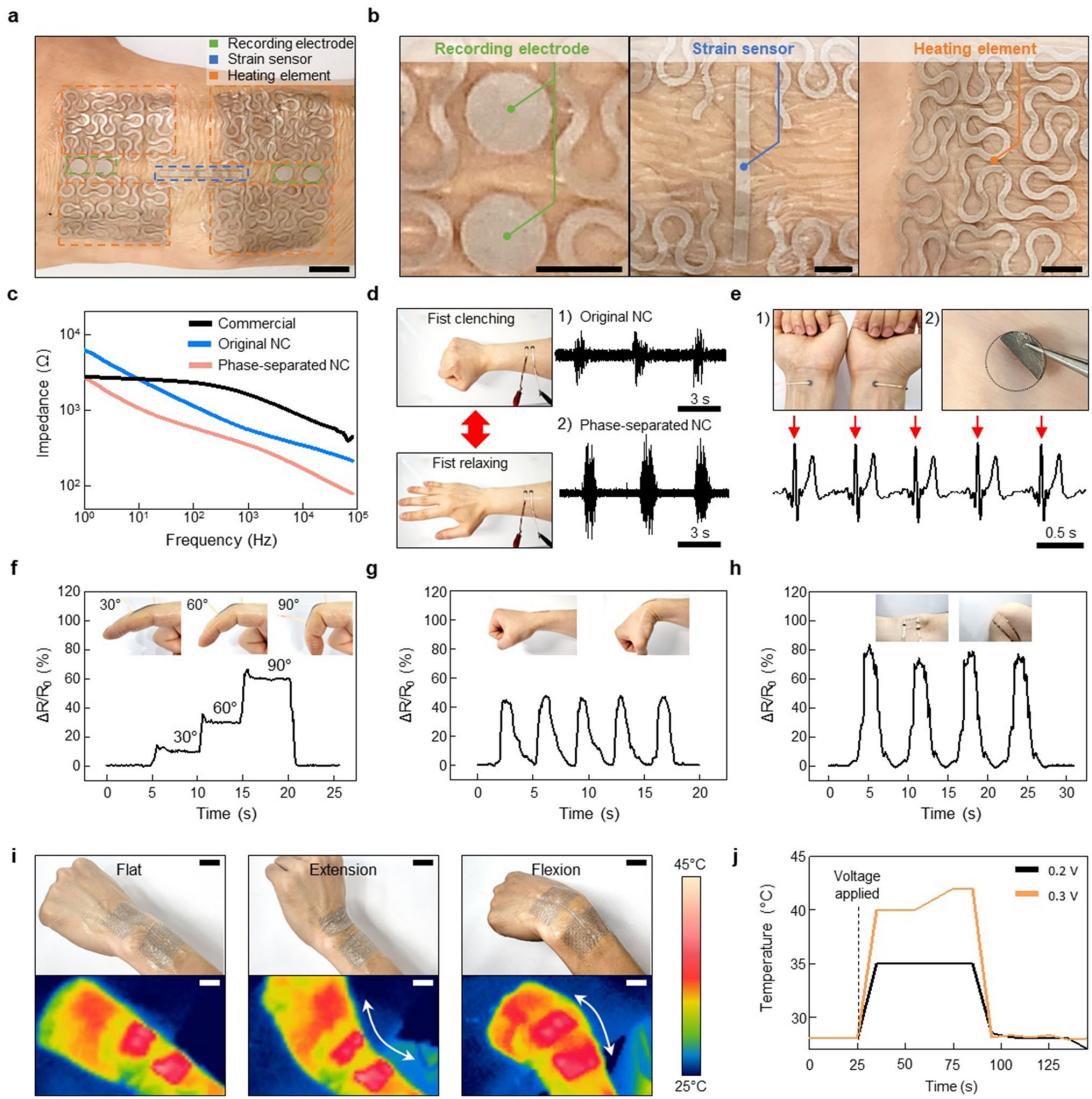
Application of the phase-separated NC to skin electronics. (**a**) A photograph that shows versatile wearable electronic device consisting of the NC. (**b**) Enlarged photographs of (**a**): electrophysiological sensors (left), strain sensors (middle), and heating elements (right). (**c**) Impedance of the commercial electrode, original NC, and phase-separated NC at the skin–electrode interface. (**d**) EMG signals recorded with the original (1) and phase-separated (2) NCs during clenching and relaxing motion. (**e**) NC electrodes affixed to inner wrists for the ECG measurement (1), no skin irritation observed after use (2), and ECG recorded with NC electrodes (bottom). (**f**)–(**h**) Resistance change during finger (**f**), wrist (**g**), and knee (**h**) flexion motions. (**i**) Optical and infrared camera images showing reliable heating performance of the NC on a wrist under flat (left), extension (middle), and flexion (right). (**j**) Temperature–time profiles with applied voltages of 0.2 V and 0.3 V. Scale bars, 1 cm (**a**); 4 mm (**b**); 2 cm (**i**).

A wearable electronic device is fabricated by patterning the NC with the laser cutting method (Fig. 5a). This device can be conformally adhered to the skin, and includes electrophysiology sensors for electrocardiogram (ECG) and electromyogram (EMG) measurement, strain sensors for capturing human motion signals, and heating elements for thermal actuation (Fig. 5b). All human experiments of this report were approved by the Institutional Review Board of Seoul National University (IRB No. 2308/002–032). Accurate measurement of electrophysiological signals requires a low interfacial impedance between the electrode and skin^43^. So, we conducted a comparative analysis of the skin-device interfacial impedance for the commercial electrode, original NC and phase-separated NC (Fig. 5c). Due to the presence of precipitated Ag NPs in the phase-separated NC, it exhibits lower impedance than both the original NC and commercial electrode. Specifically, at the EMG measurement frequency of 300 Hz, the impedance values were 2,028 Ω for the commercial electrode, 779 Ω for the original NC, and 457 Ω for the phase-separated NC (Fig. S7a). Furthermore, the phase-separated NC showed low impedance values even under various skin deformations, including twisting, stretching, poking, and bending (Fig. S7b). Lower interfacial impedance is crucial in electrophysiological signal measurements from human skin as it directly contributes to achieving a higher signal-to-noise ratio (SNR). The reduced interfacial impedance leads to enhanced signal clarity and sensitivity, thereby improving the overall quality and reliability of the recorded electrophysiological signals. This observation is particularly observed in EMG measurements. During these measurements, working and counter electrodes are attached to the volunteer's anterior forearm. Subsequently, by clenching and relaxing the fist, EMG signals are recorded (Fig. 5d, left). Immediate signals emerged upon fist clenching and disappeared upon relaxing (Fig. 5d-1, d-2). The phase-separated NC demonstrated a significantly higher SNR of around 13.92 dB. This improvement can be attributed to the reduced interfacial impedance of the phase-separated NC, outperforming the SNRs of the original NC and commercial electrode, which were approximately 6.92 dB and 6.05 dB, respectively (Fig. S7c, d). Furthermore, successful ECG recordings are achieved using the phase-separated NC electrodes affixed to both inner wrists (Fig. 5e-1). These electrodes conformally adhered to the skin, facilitating long-term measurements without skin irritation (Fig. 5e-2). ECG signals clearly exhibited P, Q, R, S, and T waves and QRS peaks are marked by red arrows (Fig. 5e, bottom). The two adjacent R peaks showed an interval of approximately 0.75 s, indicating a heart rate of 79 beats per min. Other parameters commonly used for diagnosing cardiac conditions also displayed normal values (Table S2), showing the substantial potential of the NC-based wearable sensors for effective bio-signal monitoring.

For body motion analyzing, we utilized the phase-separated NC as a resistance-type strain sensor, affixing it to fingers, wrists, and knees to enable real-time monitoring of joint motion. When the finger is flexed from a straightened state to a bent state with angles of 30°, 60°, and 90°, relative resistance change (ΔR/R_0_) are ~ 10%, ~ 30%, and ~ 60%, respectively (Fig. 5f). The resistance remained constant when the finger is stationary and promptly reverted to its initial value upon returning to a straight position. The strain sensor also accurately tracks and distinguishes the extension and flexion of larger joints such as the wrist and knee. In the case of the wrist, 40% resistance change was observed when bent (Fig. 5g), while for the knee, which bends more significantly than the wrist, the resistance changed by up to 80% (Fig. 5h). These results demonstrate that the phase-separated NC can effectively monitor joint flexion levels and repetitive bending motions.

The phase-separated NC with remarkably low contact resistance can generate sufficient and uniform heat for thermal stimulation through Joule heating even at low voltages. Exploiting this property, we fabricated a wearable heater by patterning the phase-separated NC. Afterwards, the manufactured heater was worn on the wrist, and its heating performance was evaluated while the wrist was in motion (Fig. 5i). The conformal contact was well maintained even during movements, ensuring consistent heat generation and stable temperature control. We recorded the temperature–time profile based on input voltage, and upon applying the voltage, the temperature increased within 10 s and then stabilized quickly (Fig. 5j). Furthermore, after turning off the voltage, the temperature promptly decreased, emphasizing its suitability for use as a wearable heater.

## Discussion

We presented a material strategy for a stretchable conductor which satisfies the key requirements for high-performance skin electronics, such as high conductivity, excellent stretchability, and low contact resistance, through phase separation between Ag NWs and Ag NPs within NC. The phase separation inside the NC was achieved by inducing precipitation of hydrophobically modified Ag NPs in the NC. The precipitated Ag NPs reinforce the electrical percolation network and lead to a significant enhancement of charge transfer efficiency at the bottom surface of the NC. The charge transfer efficiency was improved further by slowing down the evaporation time of the solvent from the NC solution. This enabled to achieve conductivity of ~ 18,535 S cm^−1^ and stretchability of ~ 80%. Also, average contact resistance on both top and bottom surfaces of the NC could be minimized. The phase-separated NC was successfully applied to skin electronics, effectively recording electrophysiological signals (ECG and EMG), monitoring body motion, and delivering thermal stimulation to the human skin.

## Methods

### Materials

Ethylene glycol, ethanol, and toluene were purchased from Samchun Chemicals (Republic of Korea). Poly(styrene-ethylene-butylene-styrene) (SEBS (H1041)) elastomer was obtained from Asahi Kasei (Japan). Silver nitrate, copper (II) chloride, poly(vinylpyrrolidone) (PVP, M_w_, ~ 40 k and ~ 1,300 k), 1-Propanethiol, and 3-Mercapto-1-propanol were purchased from Sigma Aldrich (USA).

### Synthesize of Ag NWs and Ag NPs

Ag NWs and Ag NPs were synthesized with slight modifications by a previously reported method^33,34^. For Ag NWs, 0.38 g of PVP (M_w_, ~ 1,300 k) was mixed with 145.6 g of ethylene glycol using a magnetic stirrer at 250 rpm and heated to 180 °C in an oil bath. Then, the magnetic stirrer was switched off, 0.8 mL of 4 mM copper (II) chloride solution was injected, followed by a precursor solution (0.48 g silver nitrate dissolved in 30 mL ethylene glycol) at 50 μL s^−1^. After the injection, the solution was kept in oil bath for 20 min, after which the oil bath was switched off to allow the resulting solution to cool to room temperature.

For Ag NPs, 1.9 g of PVP (M_w_, ~ 40 k) was mixed with 131.2 g of ethylene glycol and heated to 170 °C in an oil bath. Subsequently, the precursor solution (0.48 g silver nitrate dissolved in 30 mL ethylene glycol) was added and mixed for 10 min. Then, the solution was allowed to cool to room temperature after switching off the magnetic stirrer and oil bath. The Ag NWs and Ag NPs solutions were each washed 4 times with ethanol using a centrifuge for 10 min (Ag NWs: 2,000 rpm, Ag NPs: 13,000 rpm) and then redispersed in ethanol for further use.

### Ligand exchange

For ligand exchange, 200 mg of Ag NWs were dispersed in 70 mL of ethanol, and 400 μL of desired thiol ligand was added. Similarly, for Ag NPs, 150 mg of Ag NPs was dispersed in 35 mL of ethanol, and 400 μL of desired thiol ligand was added. After adding the thiol ligand, the solution was mixed at 500 rpm at room temperature for 2 h using a magnetic stirrer. Subsequently, the solutions were each washed 5 times with ethanol using a centrifuge for 5 min (Ag NWs: 2,000 rpm, Ag NPs: 13,000 rpm). To verify the presence of the ligand on the surface of the silver nanomaterials, the samples were analyzed using Discovery TGA (TA Instrument, USA).

### Preparation of the NC

The desired amount of Ag nanomaterials is mixed with an organic solvent (mixture of ethanol and toluene with 1:5 volume ratio) and 40 mg of SEBS polymer. Then, the NC solution is poured into a Teflon mold (2.5 cm × 2.5 cm; width × height) and dried at room temperature until fully solidified. The optimized phased-separated NC was prepared using 30 mg of Ag NWs, 15 mg of Ag NPs, and 40 mg of SEBS, and 3.9 mL of organic solvent. The optimal ratio of Ag NWs to Ag NPs was determined by comparing the conductivity and contact resistance of NCs produced under various ratio conditions (Fig. S8).

### Analyzing the structure and electrical performances of NC

Surface and thickness of the NC were observed by using Field-Emission Scanning Electron Microscopy (SUPRA 55VP, Carl Zeiss, Germany, and S-3400N, Hitachi, Japan) and Focused Ion Beam (AURIGA, Carl Zeiss, Germany).

Electrical performances of the NC were assessed by measuring its conductivity, contact resistance and interfacial impedance. Conductivity ($\sigma$) was calculated using the equation: $\sigma =l / (R\bullet w\bullet t)$ (*l*: length, *R*: resistance (Ω), *w*: width, t: thickness). For the measurement, the NCs were cut into rectangles (20 mm $\times$ 7 mm), and copper (Cu) foil strips (40 mm $\times$ 2 mm) were attached to the NC with an equal interval of 3 mm. Contact resistance was measured using the transmission line method^44^, and sheet resistance was determined using a 4-point probe method (Keithley 2400, Tektronix, USA)^45^. To measure the impedance, the volunteer’s anterior forearm was wiped with alcohol, followed by the application of conducting gel (SignaGel for electrodes, Parker). Two NC electrodes (square pattern, 1 cm × 1 cm) were mounted on the skin with a spacing of 5 mm, and Cu foil strips were attached to the electrodes for connection. Impedance was measured in the frequency range of 1 ~ 10,000 Hz using an electrochemical analyzer (CHI660E, CH Instruments).

### Comparison of LED brightness of the original and phase-separated NCs

The Cr/Au (7/70 nm) patterns were thermally deposited onto a glass substrate using a shadow mask. A total of 54 NC arrays (1 mm × 2 mm) were patterned on a 30:1 PDMS (Sylgard 184, Dow Corning, USA) slab using a laser cutter (VLS 2.30, Universal Laser Systems, USA). The PDMS with NC patterns was attached to the glass substrate with the Cr/Au patterns and secured with clamps to form connected Au-NC line.

Brightness of the original and phase-separated NC arrays was compared at a supply voltage of 2.3 V for the blue LED. Python was utilized for the numerical comparison of brightness. Pixel brightness data from LED images were collected and normalized into 10 levels. Subsequently, the area ratio for each normalized brightness was calculated, and the total brightness was compared by summing the product of normalized brightness and area fraction for each level.

### Application of the NC for wearable electrodes

NC was patterned into wearable strain sensors (Line; 1 mm × 2 cm), impedance sensor (Square; 1 cm × 1 cm), electrophysiology (EMG and ECG) sensors (Circle; diameter 6 mm), and heaters (Serpentine; width 0.8 mm) using a laser cutter. Cu foil was employed to connect these sensors to external electrical stimulating or signal recording equipment. The sensors were affixed to the skin using Tegaderm (3 M, USA). All human experiments of this report received approval from the Institutional Review Board of Seoul National University (IRB No. 2308/002–032). The experiments were carried out in accordance with approved guidelines, and all participants provided written informed consent before the experiments.

#### Wearable strain sensor

Real-time resistance measurements were performed using a source meter (2450, Keithley, USA) to capture human motion signals at joints. Bending angles of 30°, 60°, and 90° were applied to the finger to observe resistance changes. The wrist was bent to ~ 70°, while the knee underwent bending at ~ 100°.

#### Electrophysiology (ECG & EMG) sensor

For ECG measurements, conducting gel was applied to the volunteer’s inner wrists, and NC electrodes were attached as working and counter electrodes. An Ag/AgCl electrode (2223H, 3 M, USA) served as the ground electrode on the ankle. The ECG sensor was connected to conventional data acquisition (DAQ) system, Quad Bio Amp, and Powerlab 8/35 (AD Instruments, New Zealand). ECG signals were recorded at a 1,000 Hz sampling rate using the LabChart software (AD Instruments, New Zealand), which included a bandpass filtering (1–20 Hz) and signal analysis capabilities.

For EMG measurements, NC electrodes were affixed to the volunteer's forearm with a 5 mm spacing after applying conductive gel, and the ground electrode was place on the rear hand. A bandpass filter (3–300 Hz) was used, and signals were acquired during fist clenching every 5 s. Signal-to-noise ratio (SNR) was calculated using the formula: SNR_dB_ = $20 {{\text{log}}}_{10}\left(\frac{{{\text{V}}}_{{\text{signal}}}}{{{\text{V}}}_{{\text{noise}}}}\right)$, (V_noise_: the sEMG signal obtained from the settling trial, V_signal_: the sEMG signal during fist clenching). Both signals were determined using root mean square calculations.

#### Wearable heater

The NC-based heater was affixed to the wrists and connected to power supplier (U8031A, Keysight Technologies, USA) to generate the heat. A specific voltage was applied to the heater and an IR camera (FLIR i5, FLIR systems, USA) was used to measure the resulting temperature increase of the device.

### Supplementary Information

Supplementary Information.

## Data Availability

All data generated or analyzed during this study are included in this published article and its supplementary information files. The datasets used and/or analyzed during the current study are available from the corresponding author upon reasonable request.

## References

[1] Lee S (2020). Nanomesh pressure sensor for monitoring finger manipulation without sensory interference. Science.

[2] Kim DW (2022). Electrostatic-mechanical synergistic in situ multiscale tissue adhesion for sustainable residue-free bioelectronics interfaces. Adv. Mater..

[3] Koo JH (2020). Unconventional device and material approaches for monolithic biointegration of implantable sensors and wearable electronics. Adv. Mater. Technol..

[4] Min H (2022). Tough carbon nanotube-implanted bioinspired three-dimensional electrical adhesive for isotropically stretchable water-repellent bioelectronics. Adv. Funct. Mater..

[5] Gong S (2023). Hierarchically resistive skins as specific and multimetric on-throat wearable biosensors. Nat. Nanotechnol..

[6] Faiz S, Kim HW, Oh J, Veerapandian S, Jeong U (2023). High-precision stretchable ionic temperature sensor passivated with a liquid metal/block copolymer multilayer film. ACS Appl. Mater. Interfaces.

[7] Sunwoo SH (2023). Ventricular tachyarrhythmia treatment and prevention by subthreshold stimulation with stretchable epicardial multichannel electrode array. Sci. Adv..

[8] Guan YS (2022). Elastic electronics based on micromesh-structured rubbery semiconductor films. Nat. Electron..

[9] Cho KW (2022). Soft bioelectronics based on nanomaterials. Chem. Rev..

[10] Sanchez-Botero L, Agrawala A, Kramer-Bottiglio R (2023). Stretchable, breathable, and washable fabric sensor for human motion monitoring. Adv. Mater. Technol..

[11] Jiang Z (2022). A 1.3-micrometre-thick elastic conductor for seamless on-skin and implantable sensors. Nat. Electron..

[12] Lee S (2019). Ultrasoft electronics to monitor dynamically pulsing cardiomyocytes. Nat. Nanotechnol..

[13] Koo JH (2023). A vacuum-deposited polymer dielectric for wafer-scale stretchable electronics. Nat. Electron..

[14] Lyu Q (2022). A soft and ultrasensitive force sensing diaphragm for probing cardiac organoids instantaneously and wirelessly. Nat. Commun..

[15] Lu B (2019). Pure PEDOT: PSS hydrogels. Nat. Commun..

[16] Feig VR, Tran H, Lee M, Bao Z (2018). Mechanically tunable conductive interpenetrating network hydrogels that mimic the elastic moduli of biological tissue. Nat. Commun..

[17] Zhou T (2023). 3D printable high-performance conducting polymer hydrogel for all-hydrogel bioelectronic interfaces. Nat. Mater..

[18] Liu S, Shah DS, Kramer-Bottiglio R (2021). Highly stretchable multilayer electronic circuits using biphasic gallium-indium. Nat. Mater..

[19] Wang S (2022). Intrinsically stretchable electronics with ultrahigh deformability to monitor dynamically moving organs. Sci. Adv..

[20] Ma Z (2021). Permeable superelastic liquid-metal fibre mat enables biocompatible and monolithic stretchable electronics. Nat. Mater..

[21] Lee Y (2021). Standalone real-time health monitoring patch based on a stretchable organic optoelectronic system. Sci. Adv..

[22] Liu Y (2019). Soft and elastic hydrogel-based microelectronics for localized low-voltage neuromodulation. Nat. Biomed. Eng..

[23] Veerapandian S (2021). Hydrogen-doped viscoplastic liquid metal microparticles for stretchable printed metal lines. Nat. Mater..

[24] Park M (2012). Highly stretchable electric circuits from a composite material of silver nanoparticles and elastomeric fibres. Nat. Nanotechnol..

[25] Matsuhisa N (2017). Printable elastic conductors by in situ formation of silver nanoparticles from silver flakes. Nat. Mater..

[26] Jiang Z (2019). Highly stretchable metallic nanowire networks reinforced by the underlying randomly distributed elastic polymer nanofibers via interfacial adhesion improvement. Adv. Mater..

[27] Guan YS (2020). Air/water interfacial assembled rubbery semiconducting nanofilm for fully rubbery integrated electronics. Sci. Adv..

[28] Lim C (2022). Facile and scalable synthesis of whiskered gold nanosheets for stretchable, conductive, and biocompatible nanocomposites. ACS Nano.

[29] Yun YS, Kim DH, Kim B, Park HH, Jin HJ (2012). Transparent conducting films based on graphene oxide/silver nanowire hybrids with high flexibility. Synth. Met..

[30] Jung D (2023). Metal-like stretchable nanocomposite using locally-bundled nanowires for skin-mountable devices. Adv. Mater..

[31] Kim HJ (2023). Integration of conductive nanocomposites and nanomembranes for high-performance stretchable conductors. Adv. Nanobiomed Res..

[32] Sunwoo SH (2023). Stretchable low-impedance conductor with Ag-Au-Pt core-shell-shell nanowires and in situ formed Pt nanoparticles for wearable and implantable device. ACS Nano.

[33] Jung D (2021). Highly conductive and elastic nanomembrane for skin electronics. Science.

[34] Jung D (2022). Adaptive self-organization of nanomaterials enables strain-insensitive resistance of stretchable metallic nanocomposites. Adv. Mater..

[35] Guan Y-S, Yu C (2022). Interfacial assembly of metallic nanomembranes for highly stretchable conductors. Matter.

[36] Lin Y (2022). Highly conductive and compliant silver nanowire nanocomposites by direct spray deposition. ACS Appl. Mater. Interfaces.

[37] Choi S (2018). Highly conductive, stretchable and biocompatible Ag–Au core–sheath nanowire composite for wearable and implantable bioelectronics. Nat. Nanotechnol..

[38] Sunwoo SH (2020). Stretchable low-impedance nanocomposite comprised of Ag–Au core-shell nanowires and Pt black for epicardial recording and stimulation. Adv. Mater. Technol..

[39] Yu S, Li J, Zhao L, Wang B, Zheng H (2022). Stretch-insensitive capacitive pressure sensor based on highly stretchable CuNWs electrode. Sens. Actuator A Phys..

[40] Yu S (2022). Highly stable silver nanowire networks with tin oxide shells for freestanding transparent conductive nanomembranes through all-solution processes. Chem. Eng. J..

[41] Shim HJ, Sunwoo SH, Kim Y, Koo JH, Kim DH (2021). Functionalized elastomers for intrinsically soft and biointegrated electronics. Adv. Healthcare Mater..

[42] Choi S (2015). Stretchable heater using ligand-exchanged silver nanowire nanocomposite for wearable articular thermotherapy. ACS Nano.

[43] Yuk H, Lu B, Zhao X (2019). Hydrogel bioelectronics. Chem. Soc. Rev..

[44] Slewa L (2015). Transmission line method (TLM) measurement of (metal/ZnS) contact resistance. Int. J. Nanoelectron. Mater..

[45] Naftaly M (2021). Sheet resistance measurements of conductive thin films: A comparison of techniques. Electronics.

