# Supplementary material for: Phase-separated stretchable conductive nanocomposite to reduce contact resistance of skin electronics
Source: Sci Rep. 2024 Jan 16;14:1393. doi: 10.1038/s41598-024-51980-1 (PMC10791646; doi:10.1038/s41598-024-51980-1)
Supplement: Supplementary file 1 — Supplementary Information. [file 41598_2024_51980_MOESM1_ESM.pdf]

Supplementary Information

Phase-separated stretchable conductive nanocomposite to reduce contact resistance of skin electronics

Hyunjin Lee[1,2†], Hye Jin Kim[1,2†], Yoonsoo Shin[1,2†] & Dae-Hyeong Kim[1,2,3*]

[1]Center for Nanoparticle Research, Institute for Basic Science (IBS), Seoul 08826, Republic of Korea.

[2]School of Chemical and Biological Engineering and Institute of Chemical Processes, Seoul National University, Seoul 08826, Republic of Korea.

[3]Department of Materials Science and Engineering, Seoul National University, Seoul 08826, Republic of Korea.

†These authors contributed equally to this work.

*To whom correspondence should be addressed.

E-mail: dkim98@snu.ac.kr

Supplementary Figures

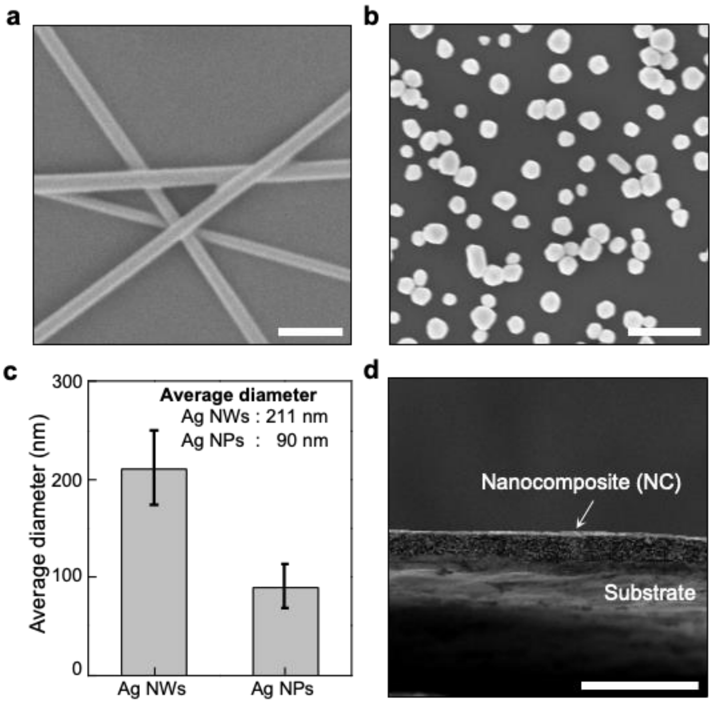

Fig. S1 | Diameters of Ag nanomaterials used for NC. a,b, SEM images of the synthesized Ag NWs (**a**) and Ag NPs (**b**). **c**, Average diameter of each nanomaterials (n = 50, mean ± standard deviation (SD)). **d**, Cross-sectional SEM image of the fabricated NC. Scale bars, 1 μm (**a**); 0.4 μm (**b**); 100 μm (**d**)

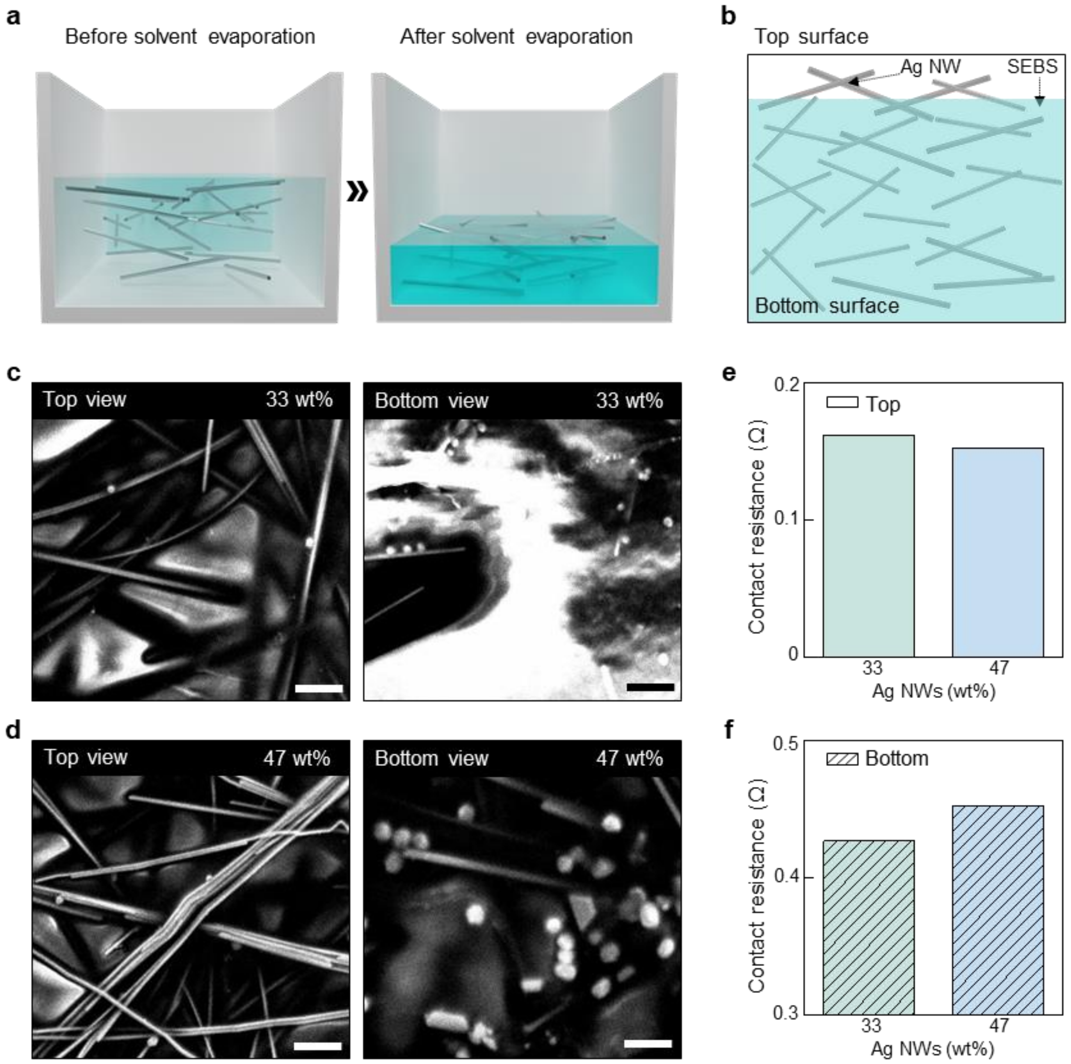

Fig. S2 | Ag NWs-based NC for the PVP ligand case. a, Sequential schematic illustrations depicting the NC fabrication process. **b**, The distribution of Ag NWs within the SEBS matrix of the fabricated NC. **c,d**, SEM images showing both top and bottom surfaces of the NCs in different weight fraction of Ag NWs. **e,f**, Contact resistances of the top (**e**) and bottom (**f**) surfaces of NC in different weight fraction of Ag NWs (Contact with copper (Cu) foil, contact area = 14 mm^2.). Scale bars, 2 μm (**c,d**)

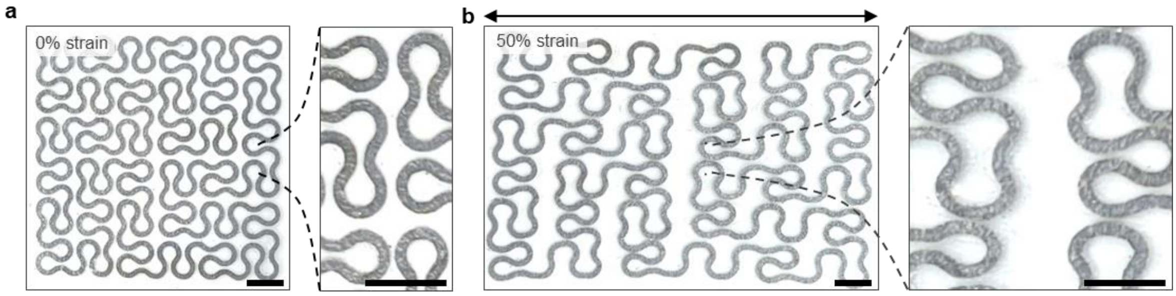

Fig. S3 | Phase-separated NC patterned using a laser cutter. a,b, Camera images illustrating the stretching performance of NC under different conditions: No strain (**a**) and 50% strain (**b**). Scale bars, 5 mm

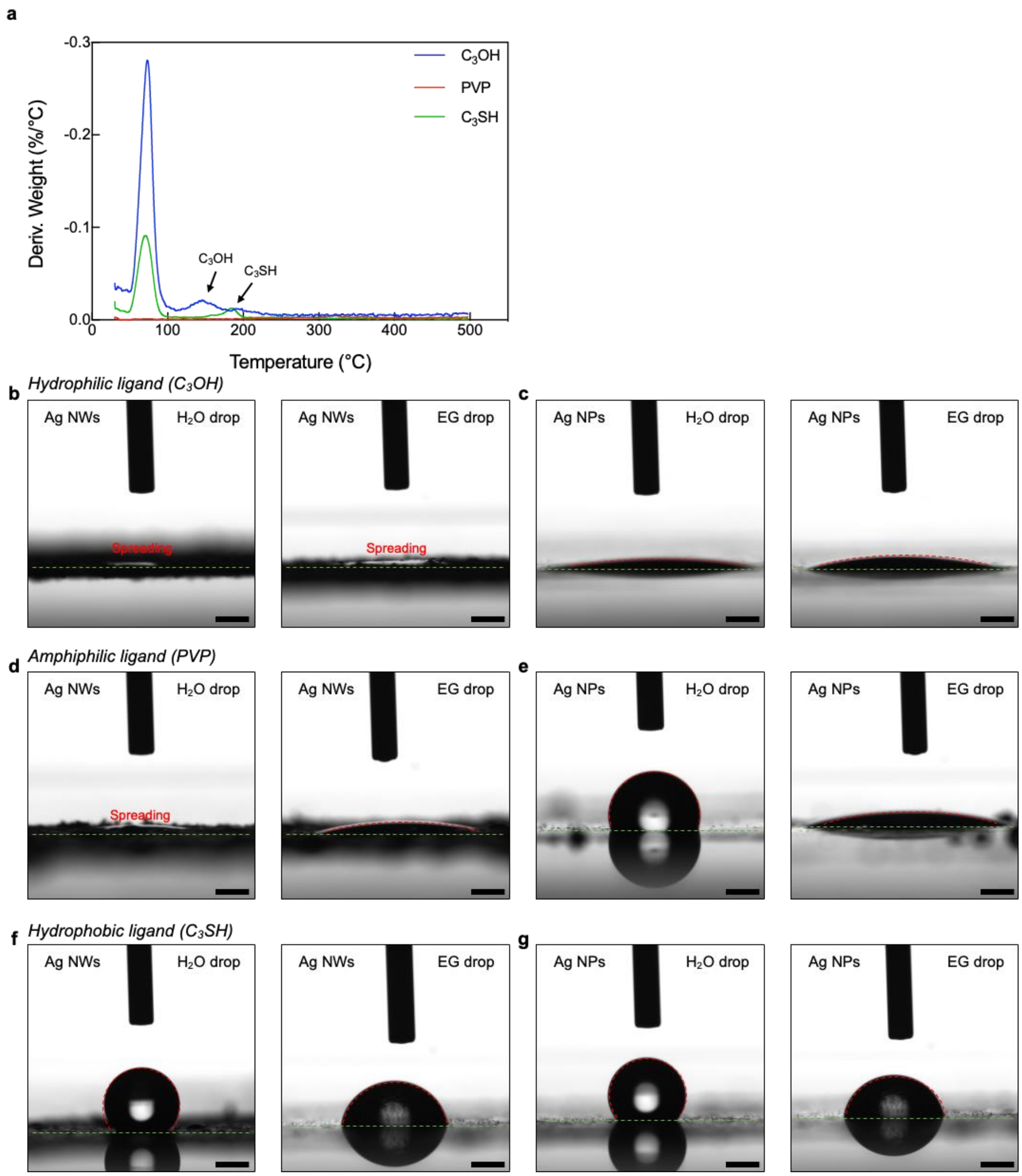

Fig. S4 | Surface properties analysis of nanomaterials. a, TGA results of nanomaterials with surface ligand modifications. **b-g**, Contact angle measurements of Ag nanomaterials. Image of DI water (H_2O) and Ethylene glycol (EG) droplets on the films of nanomaterials with various ligands. (**b**) Ag NWs with C_3OH. (**c**) Ag NPs with C_3OH. (**d**) Ag NWs with PVP. (**e**) Ag NPs with PVP. (**f**) Ag NWs with C_3SH. (**g**) Ag NPs with C_3SH. Scale bars, 1 mm.

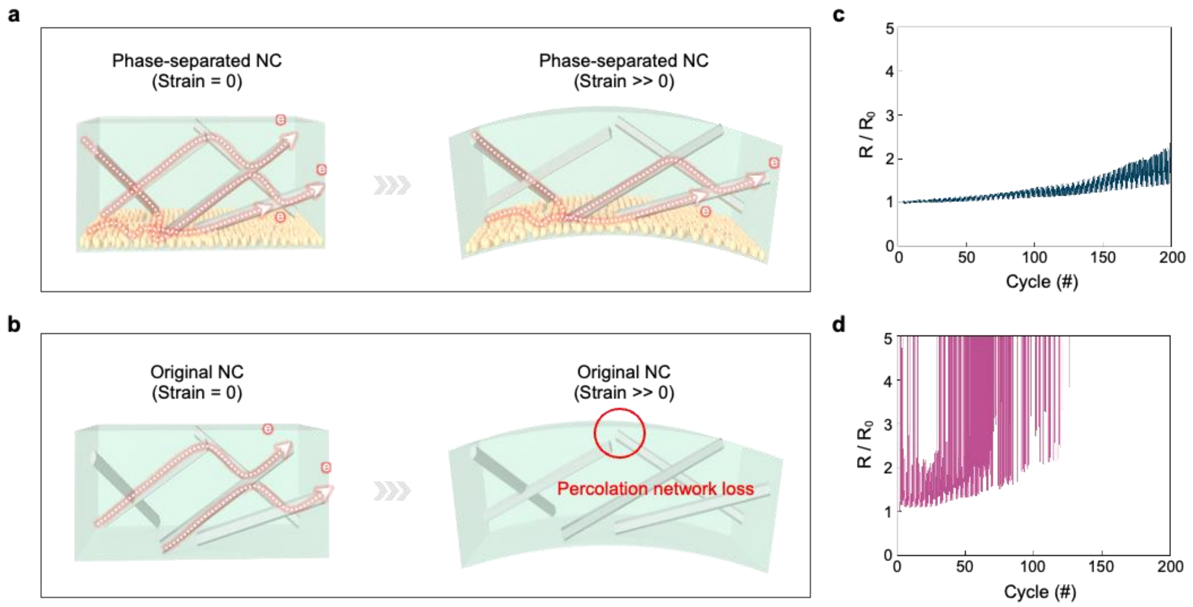

Fig. S5 | Electrical percolation network and material performance in both original and phase-separated NCs. a,b, Schematic illustration depicting the electrical percolation network inside the NCs. (**a**) Electrical connection sustaining through the Ag NPs positioned on the bottom surface within the phase-separated NC. (**b**) Loss of electrical connection in the original NC. **c,d,** Relative resistance changes of NCs during 200 cycles of stretching. (**c**) Phase-separated NC. (**d**) Original NC.

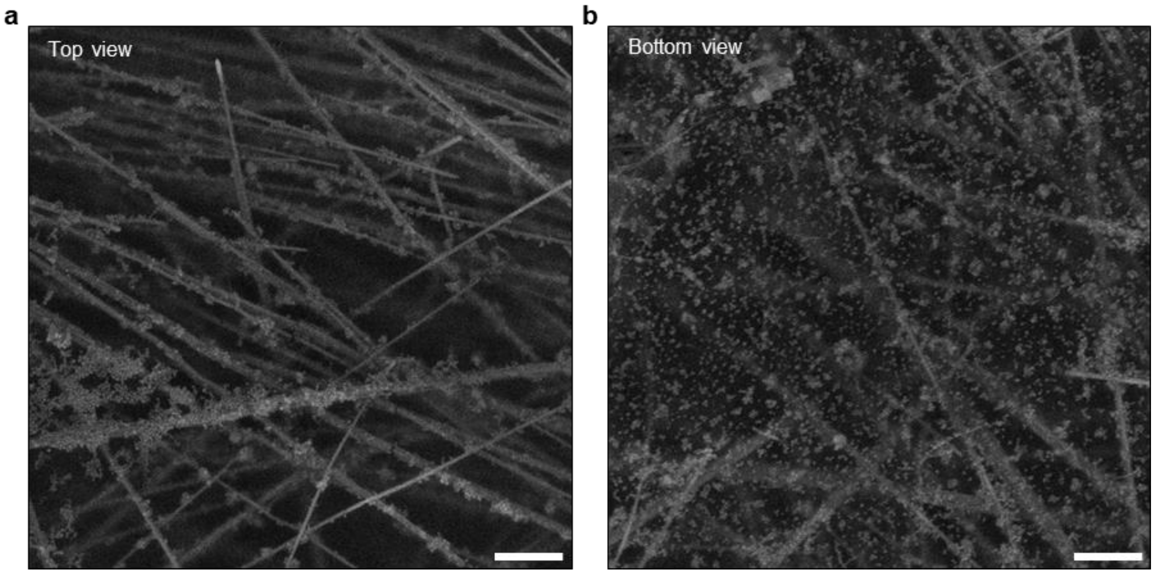

Fig. S6 | NC fabricated using a 3.0 mL solvent-containing NC solution. SEM images showing top (**a**) and bottom (**b**) surfaces of NC. Scale bars, 2 μm

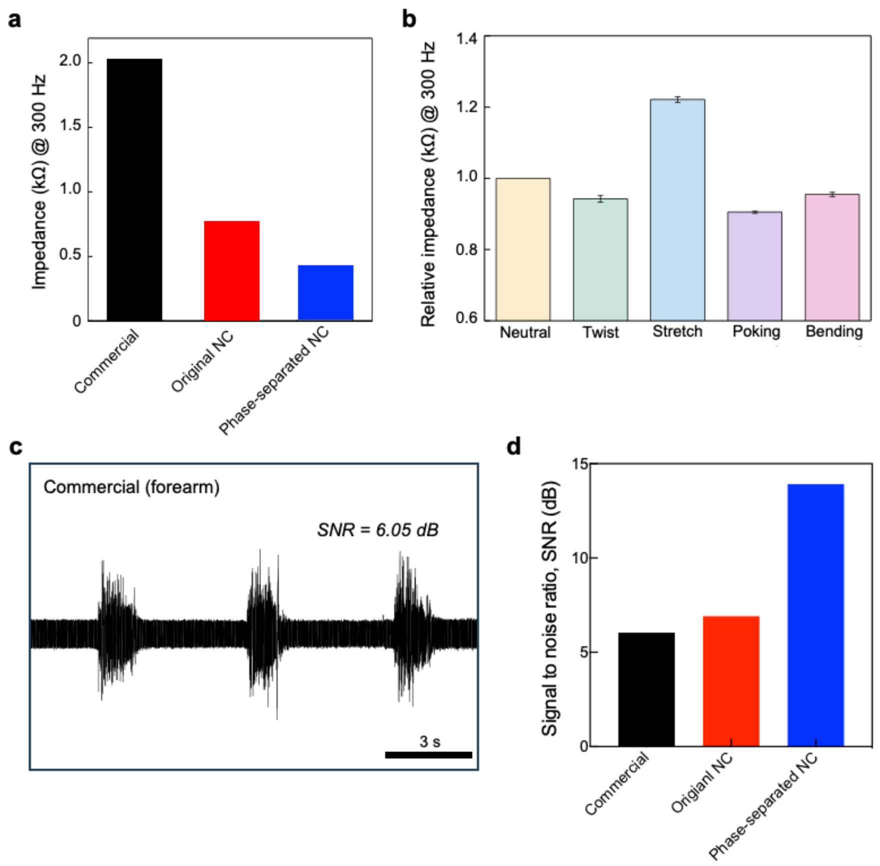

Fig. S7 | Interfacial performances of the phase-separated NC. a, Interfacial impedance values of commercial electrode, original NC, and phase-separated NC at 300 Hz. **b**, Relative impedance values of the phase-separated NC under various skin deformations, such as twisting, stretching, poking, and bending. **c**, EMG signals recorded using the commercial electrode on the forearm during clenching and relaxing motions. **d**, SNR comparison among the commercial electrode, original NC, and phase-separated NC at the skin-electrode interface.

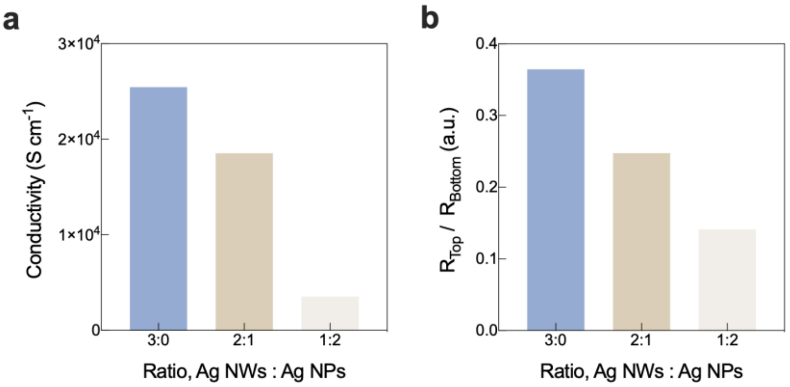

Fig. S8 | Materials performances of phase-separated NC according to the ratio of Ag NWs to Ag NPs. a, Conductivities **b,** Relative contact resistances (R_{Top} / R_{Bottom})

Supplementary Tables

Tab. S1. Contact angle of DI water (H_2O) and Ethylene glycol (EG) droplets on surfaces coated with Ag NWs and Ag NPs, varying with different ligand types.

Ligand	Nanomaterials	Contact angel of H_2O (°)	Contact angel of EG (°)
C_3OH	Ag NWs	–	–
	Ag NPs	15.1	16.2
PVP	Ag NWs	–	22.1
	Ag NPs	107.1	20.7
C_3SH	Ag NWs	137.5	80.2
	Ag NPs	129.3	79.5

Tab. S2. Key parameters of recorded ECG compared with normal range

Parameters	Recorded ECG	Normal range
PR interval (s)	0.7575	0.6 – 1.2
Heart rate (BPM)	79.21	60 – 100
PR interval (s)	0.1203	0.12 – 0.20
P duration (s)	0.07895	~ 0.08
QRS interval (s)	0.10	0.07 – 0.10
QT interval (s)	0.3128	~ 0.44
QTc (s)	0.3594	~ 0.43
